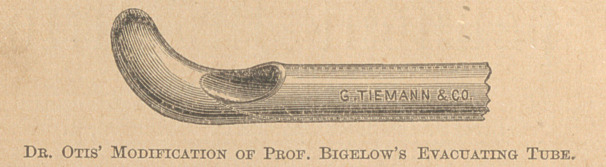# Removal of Stone from the Bladder by “Lithotrity at a Single Sitting”

**Published:** 1883-01

**Authors:** F. N. Otis

**Affiliations:** Clinical Professor in the College of Physicians and Surgeons, New York


For Contents See Page 64.

THE

### Independent Practitioner.

Vol. IV. January, 1883. No. 1.

### Original Communications.

We are not responsible for the opinions expressed by contributors.

Article I.

### REMOVAL OF STONE FROM THE BLADDER BY "LITHOTRITY AT A SINGLE SITTING" (BIGELOW'S OPERATION), COMPLICATED BY AN ENCYSTED STONE, WHICH SUBSEQUENTLY GAVE FARTHER AND CHARACTERISTIC TROUBLE.

BY F. N. OTIS, M. D.

Clinical Professor in the College of Physicians and Surgeons, New York.

Mr. E., merchant, aged 58, had suffered for over a year previous with frequent and more or less painful urination. Urine passed every two hours during the day and once or twice at night: pain and frequency both aggravated by riding in wagon, or on horseback. The stream stopped suddenly at times, but at other times it was large and free. Examination of the urine showed a plentiful deposit of pus, but no blood or evidence of kidney trouble. Examination of the rectum showed slight enlargement of the prostate.

In examination of the bladder, with Thompson's searcher, a stone was struck. A small lithotrite was then introduced to measure the size of the calculus. On opening the instrument at the *bas-fond* of the bladder, and then closing, a stone was grasped at five-eighths inch, and on moving the lithotrite (still grasping the stone), another stone was struck. The size of this second stone was not then ascertained.

At 3 p. m. of the same day (it having been determined to remove the stones by "rapid lithotrity at a single sitting"), the patient was etherized by Dr. Vermilyea; assistants in the operation, Drs. L. B. Bangs and J. W. Swasey. Dr. B. W. Dudley, of Lexington, Ky., was also present. The first grasp of the lithotrite was at a little over half an inch. Three crushings only were made, when the fragments seemed so small that aspiration was determined upon. This was effected by the intro-

DR. OTIS' MODIFICATION OF PROF. BIGELOW'S EVACUATING TUBE.

duction of my modification of Bigelow's evacuating tube 31. f,* and with Bigelow's aspirator the *debris* was soon and completely removed. The calculus was evidently recent and phosphatic, in amount about a drachm. This apparently disposed of one of the stones. The lithotrite was again introduced, and after a prolonged search only a fragment, about a third of an inch in diameter, could be found. Two more crushings effectually disposed of this, and the question arose as to what had become of the second calculus that was certainly present at the preliminary examination. It was, however, finally struck on the right side of the bladder, about an inch or so above the neck, but no coaxing could engage it in the jaws of the lithotrite. Various expedients were tried in various positions of the patient, but the stone, which was distinctly and easily felt in one place only, and that the one above referred to, could not in any way be coaxed into the jaws of the lithotrite. The conclusion was therefore irresistible that the stone was encysted, or imbedded in the walls of the bladder at that point, and could not be evacuated with the means then at our command.

Recovery from the operation occurred in this case without constitu-

* Prof. Bigelow has given preference to straight evacuating tubes in place of those more or less curved, is more effective in removing calculus material. I have frequently verified this in my own experience; but I have also found a greater difficulty in entering the bladder with them, especially in cases complicated with enlarged prostate. In such cases it has usually been necessary to introduce a finger into the rectum before the prostatic urethra could be traversed. Inorder to reduce the friction from introduction of instruments to the minimum, I have modified the straight evacuating tube of Prof. Bigelow by adding a small curved projection, which with all the advantage of the former I have found more easy of introduction, and hence less likely to be a source of obstruction and consequent irritation in entering the bladder, and also to prevent in greater degree the closure of the opening in the tube, from the engagement of the mucous membrane in it during the progress of the evacuation.—From "Transactions of International Medical Congress—Discussion on Recent Advances in the Methods of Extracting Stone from the Bladder." Vol. II. Page 322.

tional disturbance of any sort. Urination decreased in frequency, and by the third day after the operation the patient urinated not oftener than once in four hours, and without pain. He went to his home in the country on the seventh day, with entire freedom from any annoyance connected with his urinary apparatus.

On the 5th of May following (four months after the operation), the patient called, complaining again of frequent burning micturition, but no sudden stoppage of the stream. On examination with the lithotrite, and without ether, stone was at once struck on the right side, at about an inch from the vesical orifice. Instrument passed to the *bas-fond* and opened, but was closed without engaging the stone. This was repeated several times, completely rotating it, but without striking the calculus. It was then directed to the right side, and to the point where the encysted stone previously referred to had been located, and by the same maneuver the stone was engaged at half an inch, but the instrument could not be rotated, the stone being evidently attached to the wall of the bladder at this point. An easy crushing was made, and subsequently two fragments of about a third of an inch in diameter were separately picked up with the lithotrite in the region of the *bas-fond* and crushed. The *debris* was then aspirated and the bladder washed out, and subsequently no click (indicating remaining fragments) could be obtained. Again introducing the lithrotite, the calculus was struck, after some search, in the old position on the right side of the vesical neck, but careful and persistent effort, aided by a finger in the rectum, could not engage it in the jaws of the instrument. It was evidently encysted or in a sacculus of the bladder wall at this point.

Recovery from this operation also was prompt, all irritation ceasing in two or three days, and no further evidence of calculus was felt for nearly two months, when the same burning or micturition recurred with increased frequency, reducing the habitual interval of four or five hours to about two hours. Examination of urine showed a considerable catarrhal trouble, but gave no evidence of other trouble. Examination with the lithotrite found exactly the same condition as on the previous occasion. An accumulation of calculus material upon the encysted calculus had been sufficient to re-establish the vesical irritation. The same procedure for its removal which was adopted on the previous occasion was almost exactly repeated, and with almost exactly similar results.

On three subsequent occasions, at intervals varying from two to four months, the same accident has occurred, and has been relieved in the same way. There appears to be no prospect of permanent relief, except by a perineal section, through which, it seems probable, the encysted stone causing all the trouble might be reached, as in the case of J. C., operated on by me March 10, 1882, and published with illustrations in the New York *Medical Gazette* of April 8, 1882.

## Figures and Tables

**Figure f1:**